# Abdominal esophagocardiectomy with esophagogastric anastomosis, with vagal preservation and construction of a spiral anti reflux valve in the treatment of advanced megaesophagus

**DOI:** 10.1590/0100-6991e-20223222_en

**Published:** 2022-09-09

**Authors:** ORLANDO CONTRUCCI, CARLA MICAELE DE FREITAS, ELIAS JIRJOSS ILIAS, ALEXANDRE ZANCHENKO FONSECA

**Affiliations:** 1 - Universidade Santo Amaro, Cirurgia - São Paulo - SP - Brasil; 2 - Hospital Regional Sul, Cirurgia - São Paulo - SP - Brasil; 3 - Colégio Brasileiro de Cirurgiões - São Paulo - SP - Brasil; 4 - International Society for Diseases of the Esophagus - Vancouver - Canadá; 5 - Colégio Brasileiro de Cirurgia Digestiva - São Paulo - SP - Brasil; 6 - Sociedade Brasileira de Cirurgia Bariátrica e Metabólica - São Paulo - SP - Brasil

**Keywords:** Esophageal Achalasia, Esophagus, Heller Myotomy, Acalasia Esofágica, Esôfago, Miotomia de Heller

## Abstract

**Objective:**

present a new operative technique characterized by abdominal esophagocardiectomy, with esophagogastrus anastomosis, vagal preservation and spiral anti-reflux valve construction in the treatment of advanced megaesophagus in patients with severe systemic diseases, as well as its result in an initial group of 17 patients.

**Method:**

We selected 17 patients with advanced megaesophagus and comorbidities submitted to new technique. The following parameters were analyzed: age, sex, length of hospital stay, early and late complications, mortality, radiological/endoscopic aspects.

**Results:**

twelve male patients (70%) and five (30%) were operated on, with mean age of 51.5 years and mean hospital stay of 14.8 days. There was no mortality in the immediate intraoperative or postoperative period and there were no cases of postoperative fistula. During hospitalization there was one case of pulmonary atelectasis (5.8%), one of pleural effusion (5.8%), two of wall infection (11.7%) and one of urinary retention (5.8%). Discussion: We believe it to be an easy technique, made exclusively by the abdominal route, that is, without violating the thoracic cavity. Such a procedure would be beneficial in patients with advanced megaesophagus and important comorbidities, as well as in those with a history of previous surgeries.

**Conclusion:**

the technique described was easy to perform and safe, when performed by an experienced team, with low morbidity and mortality in patients with advanced megaesophagus and important comorbidities, which could increase your complications with more invasive and complex surgeries.

## RESUMO

**Objetivo:**

apresentar uma nova técnica caracterizada por esofagocardiectomia abdominal com esofagogastro anastomose, preservação vagal e construção de válvula antirrefluxo em espiral no tratamento do megaesôfago avançado de pacientes com doenças sistêmicas graves, assim como seu resultado em grupo inicial de 17 pacientes.

**Método:**

foram selecionados 17 pacientes com megaesôfago avançado e comorbidades submetidos a nova técnica. Os seguintes parâmetros analisados foram: idade, sexo, tempo de permanência hospitalar, complicações precoces e tardias, mortalidade, aspectos radiológicos/endoscópicos.

**Resultados:**

foram operados 12 (70%) pacientes do sexo masculino e 5 (30%) do sexo feminino, com idade média foi de 51,5 anos e tempo de permanência hospitalar médio de 14,8 dias. Não houve mortalidade no intraoperatório ou pós-operatório imediato e não houve casos de fìstula. Durante a internação houve um caso de atelectasia pulmonar (5,8%), um de derrame pleural (5,8%), dois de infecção de parede (11,7%) e um de retenção urinária (5,8%).

**Discussão:**

acreditamos ser uma técnica de fácil realização, feita exclusivamente por via abdominal, ou seja, sem violar a cavidade torácica. Tal procedimento seria benéfico em pacientes com megaesôfago avançado e comorbidades importantes, assim como naqueles com história de cirurgias prévias.

**Conclusão:**

a técnica descrita mostrou-se de fácil execução e segura, quando realizada por equipe experiente, com baixa morbimortalidade em pacientes com megaesôfago avançado e comorbidades importantes, o que poderia aumentar suas complicações com cirurgias mais invasivas e complexas.

## INTRODUCTION

Perhaps no pathology has been as intriguing to surgeons in recent centuries as the treatment of megaesophagus. Many controversial discussions about how to provide better treatment to patients with megaesophagus have awakened surgeons in a constant search for new techniques and better results^1^ ^-^ ^3^.

In the 19^th^ century, the only technique used consisted of a gastrostomy to feed patients in cases of severe malnutrition1. The 20^th^ century was marked by the development of several operative techniques and, currently, in the 21^st^ century, there is still no technique considered the best for the treatment of megaesophagus in its decompensated form^1^ ^-^ ^6^.

Surgical treatment of advanced megaesophagus has been challenging due to its complexity and postoperative complications, especially when the patient has severe comorbidities such as COPD, heart disease, liver disease, sequelae of lung diseases, and previous surgeries^1^ ^-^ ^4^ ^,^ ^7^.

Thus, until now, the cardiomyotomy proposed by Heller (1913) and its modifications undergone in recent years is the consensus procedure for the treatment of megaesophagus still in its compensated form, grades I II of the Ferreira Santos and Câmara Lopes classification, as well as the use of less invasive methods, such as balloon dilatations and endoscopic surgery (POEM)^4^ ^,^ ^8^ ^,^ ^16^. 

However, the decompensated forms, grades III and mainly IV of the Ferreira Santos and Câmara Lopes classification, are still a matter of controversy regarding treatment, for which techniques arose comprehending esophageal resections with mediastinal violation, and sometimes without mediastinal violation but with abdominal resections requiring several anastomoses^17^ ^-^ ^20^. These techniques show good results, with low morbidity and mortality, in specialized services and with experienced teams^9^ ^,^ ^10^ ^,^ ^21^ ^,^ ^22^.

Our concern in the search for a new technique was precisely to obtain results without violating the mediastinum, without a large number of anastomoses, with nerve preservation, and reduced surgical time, for the treatment of patients with decompensated megaesophagus (Grade III - IV) who had comorbidities such as COPD, heart disease, liver disease, pulmonary sequelae, and previous surgeries^8^ ^,^ ^9^ ^,^ ^22^ ^,^ ^23^.

## METHODS

Seventeen patients with grade III IV megaesophagus were operated on from May 1997 to June 2010, in the General Surgery Service of the Hospital Estadual Regional Sul. The patients had grade III IV megaesophagus and pre-existing diseases, these being the inclusion criteria. We excluded patients with extensive esophageal neoplasms and ulcers diagnosed during preoperative endoscopy.

The parameters analyzed were age, sex, length of hospital stay, early and late complications, mortality, radiological and endoscopic aspects, and degree of megaesophagus.

We present the data as absolute frequency and percentage for categorical variables and as mean and minimum-maximum range for continuous ones.

### Surgical technique

We performed a technique based on the modification of the ones proposed by Bier (1920)^4^, with resection of the distal segment of the esophagus and esophagogastric junction, restoring transit through an esophagogastric anastomosis, and Wangensteen (1951)^24^, with joint resection of the distal segment of the esophagus and of the proximal stomach, associated with pyloroplasty. We resected the abdominal esophagus together with the cardia region, with vagal preservation, and an esophagogastric anastomosis associated with an anti-reflux valve in a clockwise spiral at 360º, using the remainder of the gastric fundus^4^ ^,^ ^9^. 

As preoperative preparation, in addition to compensating for pre-existing diseases, nutritional support was provided through enteral nutrition and even total parenteral nutrition in the few cases where there was need for a greater nutrient supply due to previous malnutrition. Exhaustive lavage of the esophagus was performed the day before surgery with a Fouchet catheter to remove residues and avoid contamination, such as aspirations at the time of anesthesia.

The operation starts with a median xiphoumbilical laparotomy. In some cases, the triangular ligament of the liver is sectioned to allow for retraction of the liver left lobe and obtain better exposure of the esophagogastric region. The phrenoesophageal membrane is divided to allow dissection of the abdominal esophagus, which is retracted with a Penrose drain. After identifying the anterior and posterior vagus nerves, they are isolated with cardiac tape to preserve them.

In these cases, as it is a case of grades III and IV megaesophagus^9^, to rectify the esophagus we partially section the anterior muscles of the esophageal hiatus, and with delicate maneuvers, even digital ones, we release the distal part of the mediastinal esophagus, especially in cases of dolicomegaesophagus, to reduce it to the abdominal cavity and minimize postoperative stasis.

We ligate the gastric short and proximal vessels of the greater curvature to free up the region of the gastric fundus and better expose the operative field. Then, ligation of the left gastric artery is sometimes performed to facilitate the local resection, as well as the construction of the anti-reflux valve.

After this maneuver, we apply stitches to anchor the esophagus along the right and left pillars of the diaphragm and keep the organ straightened in the abdominal cavity. Then, we place an EH-40 forceps in the esophagus 6cm above the esophagogastric transition, where it is sectioned, and in the distal portion 3cm below the esophagogastric transition, from the lesser curvature towards the gastric fundus, where we perform the local resection through a linear cutting stapler (cardiectomy). The next step is a gastrotomy in the anterior wall of the stomach, through which we pass a 29mm ILS stapler, whose diameter is justified by the caliber and thickening of the esophageal wall, especially so when performing mechanical anastomoses. When the anastomosis is manual, gastrotomy is not performed. If by chance there is injury to the vagus, we advise to associate a pyloroplasty. Once the pouch is manufactured in the esophagus with the stapler anvil well positioned, the esophagogastric anastomosis is performed along the lesser curvature in a more anatomical way and in the anterior stomach wall to create an acute angle in the anastomosis and avoid greater reflux. After the esophagogastric anastomosis, a stitch connecting the stomach seromuscular layer and the esophagus adventitia is applied to the right and left sides, to support the anastomosis.

The anti-reflux valve is constructed by mobilizing the gastric fundus posterior to the esophagus in a clockwise direction to envelop the region at a 360° angle in a spiral fashion, being fixed with few support stitches in the esophagus and anteriorly in the remaining stomach itself. During surgery, an enteral tube is passed into the duodenum and is used for early refeeding. Oral feeding through a liquid diet is introduced on the third postoperative day, and the nasoenteral tube is kept until the 10^th^ postoperative day, even with the progression of oral diet, being removed after the possibility of fistula is rules out. We always perform drainage of the abdominal cavity, the drains being placed close to the esophagogastric anastomosis and exteriorized through the left flank to detect fistulas early.

. 

## RESULTS

Twelve (70%) male patients and five (30%) female ones underwent surgery. The age ranged from 28 to 59 years, with a mean of 51.5. The length of hospital stay ranged from 10 to 25 days, with a mean of 14.8. The minimum follow-up time was two months, and the maximum was 120 months. All patients underwent preoperative radiological and endoscopic studies.

Postoperatively, radiological examinations were performed (contrast study of the esophagus-stomach-duodenum, EED), which showed the passage of contrast to the remaining stomach in a satisfactory manner, with reduction in the caliber of the esophagus in 20% of the cases. Slow emptying was observed in 80% of cases. Of the most recent endoscopic controls of the seven patients who continued to be followed in the outpatient clinic, four had normal endoscopic aspects, one had erosive esophagitis, one displayed edematous esophagitis, treated clinically, and one, in addition to erosive esophagitis, had an early neoplasm of the esophagus.

**Table 1:** Age, sex, comorbidities, and degree of megaesophagus of the 17 patients operated on with the described technique, between 1997 and 2010.

Patient	Age	Sex	Comorbidities	Megaesophagus (grade)
1	28	male	Post-Heller relapse	III
2	44	female	SAH + Asthma	III
3	59	male	SAH + DM	III
4	52	male	SAH + DM + Portal Hypertension	III
5	54	male	Sequelae of pulmonary tuberculosis	IV
6	56	male	SAH + COPD	IV
7	49	male	SAH + heart disease	IV
8	54	male	SAH + COPD	III
9	57	male	DM + Asthma	III
10	45	male	Asthma	III
11	50	male	SAH + heart disease	III
12	58	male	SAH + COPD	III
Patient	Age	Sex	Comorbidities	Megaesophagus (grade)
13	58	male	Post-Heller relapse + SAH + DM	III
14	52	female	Relapse after botox and dilatation + SAH + Asthma	III
15	50	female	SAH + Rheumatoid Arthritis	III
16	54	female	SAH + heart disease	III
17	56	female	SAH + DM + COPD	III

**Table 2:** Number of patients and postoperative complications among the 17 patients operated on with the described technique, between 1997 and 2010.

N° patients	Complication
1 (5.8%)	pulmonary atelectasis
1 (5.8%)	pleural effusion
2 (11.7%)	surgical site infection
1 (5.8%)	urinary retention
1 (5.8%)	Diarrhea
1 (5.8%)	reapproach due to anastomotic stricture

**Figure 1:**
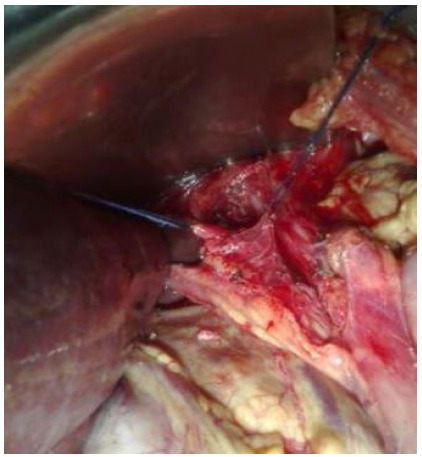
Isolated vagus nerves.

## DISCUSSION

As megaesophagus is a benign disease, which presents at different degrees in its evolution, we always ask ourselves about the best technique to be used, better long-term results, combined with lower operative morbidity and mortality^17^. As the cases in this study were grade III and IV megaesophagus with pre-existing diseases (hepatopathy, asthma, COPD, tuberculosis sequelae, SAH, heart disease) we knew that drainage surgeries would lead to lower morbidity and mortality, but earlier recurrences of symptoms, since the esophagus was dilated and/or elongated^8^. On the other hand, major resection surgeries with subtotal esophagectomy with mediastinal violation might lead to better technical results in cases of severe dolichomegaesophagus, but with surgical complications that would increase morbidity and mortality, similarly to surgeries with multiple resections and multiple anastomoses, exposing the patient to a longer operative time, increased risk of fistulas, in addition to being less physiological in terms of anatomical preservation^7^ ^,^ ^18^ ^-^ ^21^ ^,^ ^25^. 

**Figure 2:**
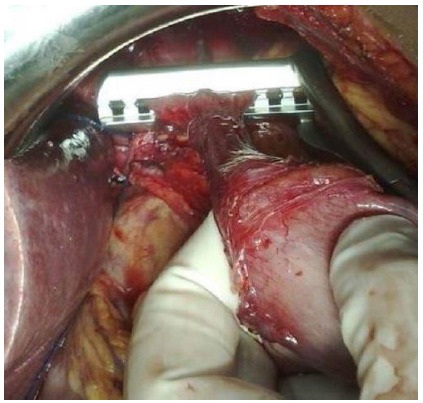
Section of the abdominal esophagus.

**Figure 3:**
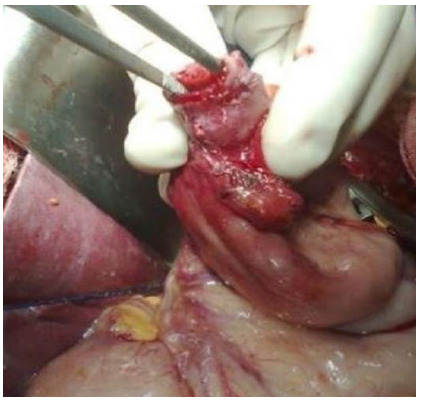
Region of achalasia.

**Figure 4:**
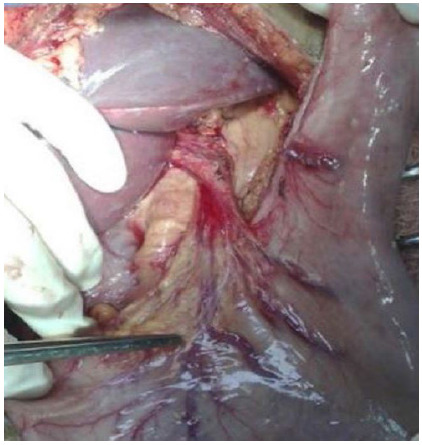
Remaining gastric fundus.

Thus, we opted for an abdominal resection technique, with a shorter surgical time and without the implications of a mediastinal or even a transthoracic approach. This is a more anatomical technique, with probable reduction of morbidity and mortality and good long-term results. In addition, preserving the vagal innervation maintains greater functionality of the digestive tract, since the pathophysiology itself promotes nerve loss in the organ (myenteric and submucosal plexus). Therefore, vagal preservation aims to help maintain gastric emptying and release of pancreatic polypeptides, which is often lost, especially in the first months of the postoperative period^17^ ^,^ ^23^. 

**Figure 5:**
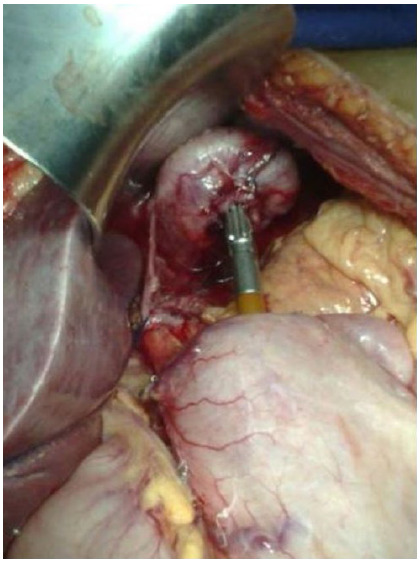
Preparation for esophagogastric stapling.

**Figure 6:**
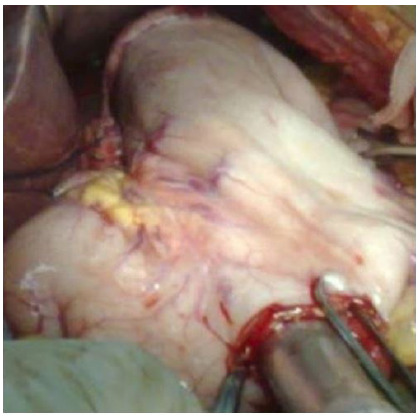
Esophagogastric mechanical anastomosis.

Esophagectomy has been placed as an option in cases of myotomy failure, as well as in cases of dolichomegaesophagus. Although studies are already underway regarding the use of myotomy and POEM, even for such advanced cases, esophagectomy is still the first choice. The technique presented, therefore, could be an option to esophagectomy in more severe patients for whom shorter surgical time is desired and myotomy and POEM are not possible, or the method has already failed. This technique has been described to reduce complications such as fistulas and anastomotic dehiscence^1^ ^-^ ^3^ ^,^ ^6^ ^,^ ^15^ ^,^ ^22^ ^,^ ^26^.

**Figure 7:**
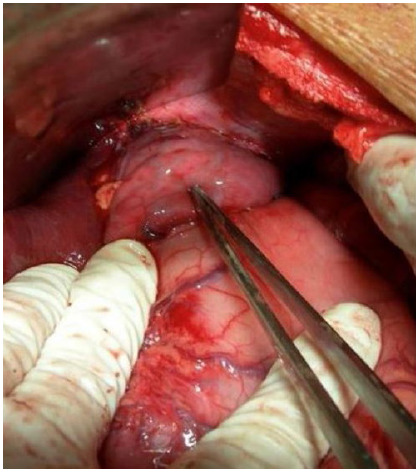
Anti-reflux valve in a clockwise spiral at 360º using the remnant of the gastric fundus.

The search continues for the best procedure for the treatment of megaesophagus, mainly its advanced forms, and this study brought one more possibility. Established methods such as Heller myotomy and less invasive methods with POEM have been the main choices for treatment of achalasia, this has been the treatment option in the study service, justifying the small sample and long time interval, since the selected patients were those whose first-choice method had failed, or would no longer have an indication for it. Thus, esophagectomy would be the choice, but the patients did not display MET for a procedure with the conventional techniques^1^ ^-^ ^3^ ^,^ ^6^ ^,^ ^22^ ^,^ ^26^ ^,^ ^27^.

Our study has some limitations such as the small sample and the wide time interval between the procedures and scientific analysis of variables, so new studies are essential for a better evaluation of the real employability of this method when compared to others. A randomized clinical trial would be the best type of study to assess the effectiveness of the technique. 

## CONCLUSION

The technique described proved to be easy to perform, in addition to being safe when performed by an experienced team, and further studies with a greater number of patients are needed. The technique has low morbidity and mortality in patients with advanced megaesophagus who have significant comorbidities, which could lead to an increase in complications when undergoing more invasive and complex surgeries.
